# Spirituality and Mental Health Care in a Religiously Homogeneous Country: Definitions, Opinions, and Practices Among Polish Mental Health Professionals

**DOI:** 10.1007/s10943-019-00911-w

**Published:** 2019-09-11

**Authors:** Edyta Charzyńska, Irena Heszen-Celińska

**Affiliations:** 1 University of Silesia in Katowice, ul. Grażyńskiego 53, 40-126 Katowice, Poland; 2 Department of Health Psychology, SWPS University of Social Sciences and Humanities, Warsaw, Poland

**Keywords:** Spirituality, Religion, Mental health professionals, Qualitative data, Content analysis

## Abstract

This qualitative study involved a sample of 121 Polish mental health professionals who were interviewed about their definitions of spirituality and their opinions and practices concerning the inclusion of clients’ spirituality in therapy. Using inductive content analysis, we identified seven categories regarding the definitions of spirituality: (1) relationship, (2) transcendence, (3) dimension of functioning, (4) a specific human characteristic, (5) searching for the meaning of life, (6) value-based lifestyle, and (7) elusiveness and indefinability. The majority of respondents claimed to include elements of spirituality in therapy. However, some participants included spirituality only under certain circumstances or conditions, or did not include it at all, citing lack of need, lack of a clear definition of spirituality, their own insufficient knowledge, lack of experience, fear, or concern over ethical inappropriateness. Implicit techniques were primarily used when working on clients’ spirituality. This article deepens the knowledge on including spirituality in mental health care, with special consideration for a specific context of a highly religious and religiously homogenous culture.

## Introduction

As a complex concept that can be expressed in varied ways, spirituality is difficult to conceptualize (Hill et al. 2000). Nevertheless, number of scholars define spirituality as a fundamental human drive for transcendent meaning, purpose, and values (Canda and Furman 2010; Koenig 2012; Pargament 2007). It is reflected in a specific, dynamic relationship of the current self with the object one considers highly valuable (e.g., God, a higher intelligence, the universe, another human, or one’s ideal self; Worthington and Aten 2009).

One of the core topics explored in studies on spirituality is its relationship to religion. Religion is defined as a shared set of beliefs, practices, and rituals related to the sacred, which are transmitted through community-based structures and organizations (Canda and Furman 2010; Koenig 2012). Although there is a considerable overlap between spirituality and religion, scholars tend to perceive them as two distinct constructs (Hill et al. 2000; Miller and Thoresen 2003). Thus, religion can be spiritual when it is well internalized and relies on a deep and personal relationship with God or a higher being, or it can be bereft of spirituality when beliefs and practices are done without meaning or engagement (Worthington and Aten 2009).

The majority of the pioneers of psychology treated clients’ spiritual beliefs and practices as expressions of psychopathology and immaturity, negating or marginalizing their role in therapy (Plante 2007). A breakthrough in the relationship between spirituality and psychology occurred at the end of the twentieth century (Miller and Thoresen 2003; Richards and Bergin 2005). This change was primarily induced by the results of many studies confirming the health-promoting role of spirituality in human life (George et al. 2000; Hill et al. 2000; Koenig 2012). Significant support for introducing spirituality into mental health care was also provided by studies of the recipients of counseling and psychotherapeutic services. These studies showed that many clients would like their spiritual issues to be addressed in treatment (Post and Wade 2014; Rose et al. 2008). Some clients feel deeply distressed because of spiritual problems or because of doubts or difficulties related to their inability to live in compliance with a certain value system (Exline and Rose 2005). Moreover, in some patients, spiritual conflicts manifest as psychopathological symptoms (Johnson and Hayes 2003). Special needs related to spiritual issues are also reported by deeply devout people. These persons often look for specialists who share their religious affiliation as they prefer therapeutic techniques that are compliant with their value system (Weld and Eriksen 2007). A meta-analysis by Worthington et al. (2011) suggested that deeply devout or spiritually committed clients may be especially receptive to incorporating religious and spiritual issues into their therapy.

### Spirituality in Mental Health Practice

In light of the studies on clients’ needs and expectations regarding the inclusion of spirituality in therapy, there is also a need to study mental health professionals’ opinions and practices on this matter. A number of studies have already investigated this topic. For instance, Hathaway et al. (2004) found that clinical psychologists believed clients’ spirituality or religiosity to be an important area of functioning that is relevant to treatment. Yet, most of these clinicians did not routinely assess spirituality and religiosity or address it in treatment planning (Hathaway et al. 2004). Quite similar results were noted by Rosmarin et al. (2013) in a study of 262 members of the Association for Behavioral and Cognitive Therapy. Less than 5% of the study’s participants believed that spiritual and religious issues are never or rarely relevant to mental health. However, in practice, almost one-fifth of the participants never or rarely inquired about or assessed their clients’ spirituality or religiosity (Rosmarin et al. 2013).

In another study (Carlson et al. 2002), 400 clinical members of the American Association for Marriage and Family Therapy (AAMFT) were asked about their beliefs on the appropriateness of addressing spiritual issues in therapy. Of the 400 surveys mailed, 153 were returned. The vast majority of the participants believed that there is a relationship between spiritual and mental health and that it is appropriate to discuss spirituality in psychological circles (96% and 84%, respectively). However, fewer participants (68%) agreed that it is appropriate for therapists to ask clients about their spirituality; even fewer believed that it is appropriate to use spiritual language in therapy (52%), to discuss a client’s spiritual symbols (48%), to help clients to develop their spirituality (42%), or to recommend spiritual programs to the client (38%). Praying with client (17%), discussing one’s own spirituality (26%), and mediating with the client (32%) were the least supported practices. Interestingly, not only does evaluation of the appropriateness of spiritual interventions decline as they become more tied to specific spiritual practices, but the same happens also for the actual use of these interventions (Cornish et al. 2012).

The results of the above studies suggest that although mental health professionals are generally positive about the idea of spirituality in therapy, they may be less supportive of initiating the topic of spirituality during the diagnostic sessions or to use explicitly spiritual techniques during therapeutic sessions. It also suggests a potential discrepancy between mental health professionals’ opinions on spirituality in therapy and their actual practices, thus indicating the need to explore them together (Frazier and Hansen 2009).

Another topic worth exploring in this context is the mental health professionals’ ways of defining spirituality. This is important because a deep reflection on one’s own understanding of the concept of spirituality is one of the requirements for providing adequate spiritual care (Daghan 2018; Vieten et al. 2016). Egan and Swedersky (2003) interviewed eight Canadian occupational therapists who acknowledged that they considered spirituality when working with clients. One of the topics of the interview was the definition of spirituality. The participants described spirituality in diverse ways, using either religious or secular language. Spirituality was perceived primarily as one’s beliefs about the world and one’s place in it and how one lives out these beliefs through reflection and conscious actions (Egan and Swedersky 2003). When describing their own beliefs and actions, the participants paid much attention to the feeling of interconnectedness of persons with different objects (themselves, other people, God, or some other transcendent force) and to the strong influence of spirituality on their own experiences and actions.

However, for some mental health professionals, spirituality is an elusive, ambiguous, or even contradictory construct (Crossley and Salter 2005). This finding is important because the ways of defining spirituality may influence mental health professionals’ therapeutic practices concerning spirituality (Hodge 2002, 2006; Ozorak 2005). In particular, the lack of definition of spirituality or the perception of it as a fuzzy concept may cause reluctance to include it in therapeutic practice (Souza 2002; Weinstein et al. 2002). Thus, it seems highly relevant to take a closer look at mental health professionals’ conceptualization of spirituality. In addition, when investigating definitions of spirituality, it is also important to explore mental health professionals’ opinions on the relationship between spirituality and religion. This is supported by the results of studies showing the different evaluations of spirituality and religion among mental health professionals: generally, they are more favorable toward the inclusion of spirituality in therapy, evaluating spiritual techniques and interventions as more appropriate than religious ones, and they claim to use the former more often than the latter (Carlson et al. 2002; Cornish et al. 2012).

### Current Study

Although over the past 20 years interest in spirituality in mental health care has grown considerably, the majority of studies on this subject among mental health professionals were carried out in religiously and spiritually diverse countries such as the USA, Canada, or Western European countries (Carlson et al. 2002; Crossley and Salter 2005; Egan and Swedersky 2003; Frazier and Hansen 2009; Hofmann and Walach 2011). The multiplicity of religions and spiritual options may have a significant influence on mental health professionals’ opinions and practices concerning spirituality in therapy. Therefore, in this study we examined the definitions of spirituality and the opinions and practices with regard to spirituality in therapy among Polish mental health professionals. Poland is a very good example of a religiously homogeneous country, with 92% of religiously oriented Poles identifying as Catholics (Central Statistical Office 2018). Although in comparison with older generations, the faith of young Poles is decreasing (Pew Research Center 2018), the study by Bullivant (2018) showed that in Poland the percentage of Catholics (82%) in the 16–29 age group is still higher than in any other of the 21 studied European countries. The high level of religiosity among Poles is the result of centuries-old tradition and turbulent history, including more than a century of occupation by other countries and a period of communism. The other important factor is the close involvement of the Church in matters of the state, along with the pontificate of the Pope John Paul II.

We deliberately focused on exploring spirituality in a highly religious country because we wanted to explore whether and how the specific context of the religiously homogenous culture affects mental health professionals’ definitions, beliefs, and therapeutic practices concerning spirituality (see Keller et al. 2013). Moreover, we decided to focus on spirituality when taking into account that the topic of religion in Polish mental health care has been explored extensively hitherto, not only by psychologists but also by theologists and pastoral counselors (e.g., Grulkowski and Głaz 2006; Płużek 2002), whereas there is a lack of studies on spirituality in these settings.

When choosing the research methodology, we were guided by the purpose of the study. We decided to apply the qualitative approach to explore the variety of opinions, beliefs, and practices among mental health professionals without restricting their responses. We believed that the qualitative research design would provide a rich description of the studied phenomenon, expressed in words embedded in the context, rather than presented only in numbers (Belzen and Hood 2006).

## Method

### Participants

The sample consisted of 121 Polish mental health professionals (85 women and 36 men). The sociodemographic characteristics of the participants and details on their professional experience are provided in Table 1.

**Table 1:** Sociodemographic and work-related characteristics of participants (*N* = 121)

Variable	*n*	%
Gender		
Female	85	70
Male	36	30
Profession*		
Psychologist	83	69
Pedagogist	8	7
Doctor	7	6
Theologist	5	4
Sociologist	4	3
Other	13	11
N/A	4	3
Psychotherapy school*		
Psychoanalytical/psychodynamic	36	30
Systemic	30	25
Gestalt	20	17
Integrative (including the Christian approach)	17 (5)	14 (4)
Cognitive behavioral	15	12
School of Addiction Psychotherapy	10	8
Humanistic/existential	7	6
Ericksonian	6	5
Other	13	11
N/A	5	4
Workplace		
Private practice	41	34
Counseling center/hospital/clinic	35	29
Private practice and counseling center/hospital/clinic	45	37
Religious denomination		
Roman Catholic	89	74
Greek Catholic	5	4
Protestant	4	3
Buddhist	4	3
Without denomination	17	14
N/A	2	2

*Some mental health professionals had more than one profession and/or graduated from more than one psychotherapy school

The mean age of the respondents was 44.2 years, SD = 10.39 (min. = 26 years; max. = 72 years). All the participants had higher education, and the majority were psychologists. The largest number of mental health professionals were psychoanalysts or psychodynamically oriented therapists (30%); the second largest group was systemic therapists (25%), and the third largest was Gestalt therapists (17%). The mean work experience as a mental health professional was 13.3 years, SD = 9.83 (min. = 1 year; max. = 44 years). The highest number among the participants (37%) had their private therapy practice and were also employed in a counseling center, hospital, or clinic. The majority of the participants (78%) identified as Catholic.

### Procedure and a Research Tool

A letter of invitation with a link to the survey was sent by e-mail to 500 Polish mental health professionals. Their e-mail addresses were obtained in two ways: by using lists available on the websites of psychological and psychiatric associations and by approaching mental health professionals using their contact information on Google. Complete responses were received from 121 mental health professionals; i.e., 24.2% of the persons who had been asked to participate in the study.

The e-mail sent to the mental health professionals informed them about the purpose of the study. The description stated that the researchers would like to study definitions, opinions and practices around spirituality, and its inclusion in therapy among Polish mental health professionals. The participants were also informed that the study was anonymous and confidential and that the results would only be used for research purposes.

The survey was made available as a Google form. Each participant completed a section that asked about their sociodemographic characteristics and the characteristics of their mental health practice. Then, participants responded to the following six questions:

- *Question 1* In your opinion, what is spirituality?
- *Question 2* In your opinion, is spirituality identical to religion? Please explain your opinion.
- *Question 3* Do you include clients’ spirituality in the diagnostic sessions? Please explain in more detail why you do this or not.
- *Question 4* Do you include clients’ spirituality in the therapeutic sessions? Please provide us with some more detail why you do this or not.
- *Question 5* If you include clients’ spirituality into your mental health practice, please describe typical techniques you use when working on the clients’ spirituality or note typical questions you ask about this topic.
- *Question 6* If you include clients’ spirituality into your mental health practice, please describe your sources of knowledge and inspiration regarding this topic.

### Approach to the Data Analysis

The responses to the questions were analyzed using qualitative methodology. To questions 1–4, we applied inductive content analysis (Elo and Kyngäs 2008), also named conventional content analysis (Hsieh and Shannon 2005). In this method, codes, categories, and subcategories are extracted from the text, without references to existing theories or previous studies. In the preparation phase of the inductive content analysis, two competent judges working independently (the first author and a psychologist familiar with the method of content analysis) read carefully through the participants’ responses to each of the four questions several times so as to find the essence of the responses (Elo and Kyngäs 2008). The unit of analysis was a part of the response referring to one discrete theme (Zhang and Wildemuth 2005). In the beginning of the organizing phase, the judges went through the text and indexed it (“open coding”) by adding codes to text segments signifying the occurrence of important information (Gläser and Laudel 2013). In the next step, after several readings of the material, the data were grouped: similar codes were combined into categories and subcategories (Elo and Kyngäs 2008; Hsieh and Shannon 2005). During the analysis, the preliminarily identified categories and subcategories were redefined whenever subsequent responses suggested the need. Then, the categories and subcategories obtained by the judges were compared to each other, and in the case of discrepancies the data were analyzed again. In such cases, two more competent judges (psychologists who had not taken part in the previous stages of analysis) were asked about their opinions, and any differences were negotiated until a consensus was achieved (Patton 1999). This work resulted in the development of the final code scheme, comprising codes, categories and subcategories, their definitions, and examples.

Further analysis of the responses to questions 1–4 was performed by two new competent judges, psychologists who had not yet taken part in the study at any stage. Their task was to encode the responses to each question according to the code scheme described above. The judges worked separately and independently. Agreement between judges was assessed using Cohen’s kappa (*κ*) coefficient. As the obtained values ranged from .71 to .79, the interrater reliability was satisfactory (Mayring 2000). Calculations were done using IBM SPSS version 24.0 (IBM Corp 2016).

For questions 5 and 6, which asked the respondents to provide examples of techniques and sources of knowledge and inspiration concerning spirituality in mental health care, the analysis was simplified compared to the analysis for questions 1–4. After preliminary careful reading of all responses to these questions, the techniques and sources of knowledge and inspiration listed by the participants were grouped under categories on the basis of their similarity (Burnard 1991; Dey 1993). The categorization process was performed independently by the first author and a psychologist who knew the procedure but did not know the purpose of the study. Any differences in assessment were discussed until all responses could be consistently attributed to the established categories.

### Trustworthiness of the Study

As suggested by Lincoln and Guba (1985), we took several steps to ascertain the trustworthiness of the study. First, to bolster its credibility, we tried to diversify our sample in terms of gender, age, and professional background. In this way, we aimed to obtain various definitions, viewpoints, and practices concerning spirituality in order to construct a rich picture of the studied phenomenon (Shenton 2004). Second, we reported a substantial number of quotes from the participants to support the transferability of the study results. This was done because we aimed not only to describe the participants’ definitions, opinions, and practices but also to provide context for their responses so that they would become meaningful to the reader (Korstjens and Moser 2018). Third, to ensure reflexive research design, and in this way improve the confirmability of the study, we included multiple investigators with expertise in qualitative research (Patton 1999). This extensive cooperation gave us the opportunity to engage in critical self-reflection about ourselves as researchers and to reveal the biases and pre-assumptions that we brought to the study. Lastly, we ensured the dependability of the study by providing a detailed methodological description, allowing the reader to replicate the study (Shenton 2004).

## Results

**Fig. 1:**
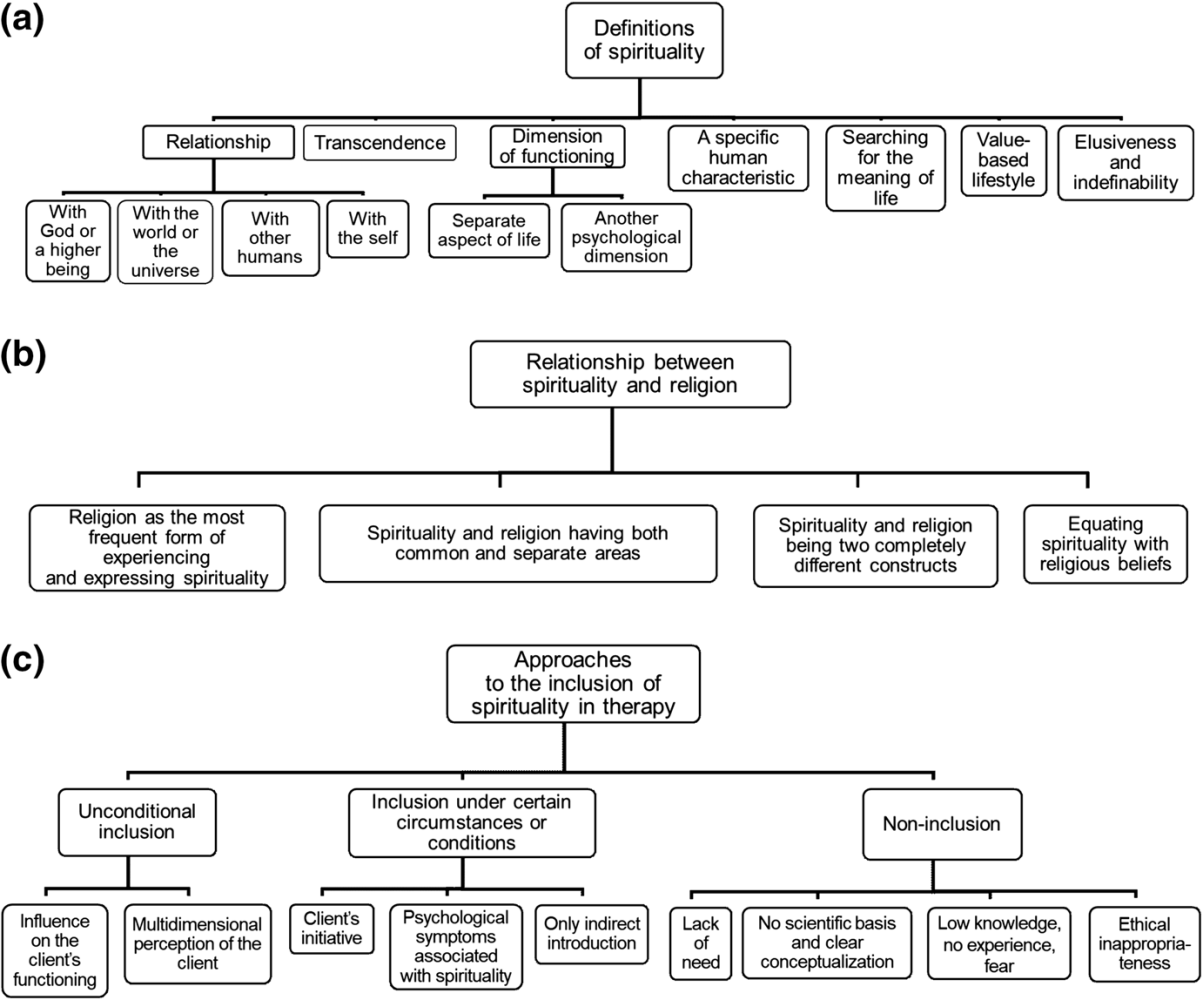
Tree codes for the participants’ responses concerning their: **a** definitions of spirituality, **b** opinions on the relationship between spirituality and religion, and **c** approaches to the inclusion of spirituality in mental health practice

All the responses quoted below are accompanied by the mental health professional’s gender (F: female; M: male) and age. The first author and a professional English translator translated the participants’ responses, which were then proofread by a native English speaker. Figure 1 shows tree codes illustrating the hierarchical structure of the data for questions 1–4 (Morse and Field 1995).

### Defining Spirituality (Question 1)

The inductive content analysis of the responses to question 1 concerning the definitions of spirituality led to the identification of seven categories: (1) relationship, (2) transcendence, (3) dimension of functioning, (4) a specific human characteristic, (5) searching for the meaning of life, (6) value-based lifestyle, and (7) elusiveness and indefinability. For “relationship” and “dimension of functioning” categories, subcategories were also identified (Fig. 1).

Participants often defined spirituality in terms of a relationship, using one of the four types of object (subcategories): (1) God or a higher being, (2) the world or the universe, (3) other humans, and (4) the self. For those respondents, the essence of spirituality is experiencing the sense of being a part of a larger and more important entity than an individual being:

For me, spirituality is a bond or relationship between the human and God (the Absolute, the Fate, the Universe, etc.), people and the environment. (F, 41)

The participants also often mentioned the transcendent character of spirituality, emphasizing that it allows the person to go beyond the limitations of mortal existence:

The quality that allows a human to overcome their material, bodily, and transient aspects. (M, 47)

Another category that emerged from the responses was spirituality understood as a dimension of functioning; this category was divided into two subcategories. Some mental health professionals defining spirituality as a dimension of functioning understood it as a separate, independent aspect of life, while others identified spirituality as another psychological dimension (Fig. 1):

The first idea: it is a fourth dimension, equally important as the biological, psychological, and social one. (F, 33)

One of the dimensions of human psyche, concerning morality, conscience, philosophy of existence, and most of all, metaphysics. (M, 54)

For some respondents, spirituality meant a specific human characteristic: for example, the ability to “love” (F, 46), “reflect” (F, 37), “admire people and the world” (F, 37), or “distinguish between the sacred and the profane” (F, 46).

Spirituality was also defined as the search for the meaning of life. According to the mental health professionals who defined spirituality this way, the need for meaning manifests itself through asking questions about the origin of humans and the purpose and meaning of human existence. They also emphasized that the choice of a particular path of spiritual growth helps create a coherent vision of reality, introducing order and harmony to life:

Spirituality is a pursuit of one’s meaning of life and trying to act accordingly. It is connected with the attempt to answer the questions of where we come from, who we are, where we are going, and what we live for. It includes both theory, that is, looking for a vision to organize our life, and practice, which means engagement in specific spiritual practice to make our life meaningful. (M, 49)

Spirituality was also perceived as a value-based lifestyle. This kind of value system is characterized as absolute, timeless, and culture-independent:

Making one’s life richer and more profound thanks to timeless and general human values, applying them in one’s life. (F, 58)

A few mental health professionals pointed to the elusiveness of spirituality and problems with defining it. They stressed that the concept is blurred and less precise than religion. In their opinion, spirituality is interpreted by each person individually, using non-rational factors such as intuition:

I don’t know any satisfactory, precise definitions. I have the impression it is an obscure concept which each person understands in her or his own way. (M, 49)

### Relationship Between Spirituality and Religion (Question 2)

The next question asked the mental health professionals to express their opinion on the relationship between spirituality and religion. Four categories were identified on the basis of the participants’ responses: (1) religion as the most frequent form of experiencing and expressing spirituality; (2) spirituality and religion having both common and separate areas; (3) spirituality and religion being two completely different constructs; and (4) equating spirituality with religious beliefs (Fig. 1).

The majority of the respondents treated religion as a form of spirituality. For those mental health professionals, religion is the most frequent form of experiencing and expressing spirituality. At the same time, they paid attention to the fact that spirituality can also occur in a non-religious context:

Non-religious spirituality is possible, e.g., looking for transcendence and inspiration in existential dilemmas. Personally, I represent religious spirituality, but I can also see other ways to experience it. (M, 71)

Another frequent opinion was that spirituality and religion have both common and separate areas. Just like the mental health professionals mentioned above, these respondents thought religion was one, but not the only, expression of spirituality. In their opinion, religion without a spiritual element is also possible. Such a religion would include the instrumental celebration of religious rites in order to satisfy one’s needs and to avoid the unpleasant emotional and social consequences of failure to observe religious principles:

From my experience, people who declare they are not religious sometimes have even more developed spirituality than people who belong to a religion. On the other hand, people who claim to be deeply devout sometimes have very shallow spirituality, limited to fear of breaking religious rules. (M, 41)

For some mental health professionals, religion and spirituality are two completely different concepts. Those participants highlighted the external, institutionalized, and routine character of religion, reducing it to a cultural phenomenon. By contrast, they emphasized the personal, internal, and intimate nature of spirituality:

I understand religion in the context of a ritual, traditional, and cultural behavior, and spirituality as a personal, vivid experience of searching for the meaning. (M, 37)

For few participants, spirituality was equated with religious beliefs:

It’s actually the same. When clients refer to the topic of spirituality, they talk about their religious beliefs and values. (F, 31)

### Inclusion of Spiritual Issues in Diagnostic and Therapeutic Sessions (Questions 3 and 4)

The next two questions explored whether and for what reasons mental health professionals included elements of spirituality during diagnostic and therapeutic sessions. Three main categories were identified for both questions: (1) unconditional inclusion, (2) inclusion under certain circumstances or conditions, and (3) non-inclusion. For all three categories, subcategories were determined, explaining why mental health professionals included or did not include spiritual topics in diagnostic and therapeutic sessions (Fig. 1).

Many participants stated that they included the subject of spirituality in the diagnostic and therapeutic sessions, and some did this for each client. Participants who included the issues of spirituality in their diagnostic and therapeutic sessions unconditionally provided two reasons for doing so: first, they highlighted that spirituality has an influence on the client’s functioning, well-being, and recovery. Second, they pointed out that including this aspect makes it possible to perceive the client multidimensionally, giving them an opportunity to understand the client in a deeper and more holistic way:

I include it because I want to know how active this area is in the patient’s life. I want to know if I can refer to it and to what extent. I assume the spiritual area affects other levels of human functioning very much: it directly influences their mental life, but also physical functioning. (F, 52)

I include it, because without it, my perception and understanding of the client would be incomplete. (F, 68)

Many mental health professionals stated that they included the subject of spirituality in diagnostic and therapeutic sessions only under certain circumstances or conditions. The most frequently mentioned situation in which spiritual issues would arise in treatment was that the client brought up the subject first. These mental health professionals emphasized that if they initiated this subject themselves, it could be perceived as repressive or even humiliating by clients with a low level of spirituality or those for whom it is not an important value:

The willingness to speak about it should be expressed by the patient first, because people who cannot find spirituality in themselves may feel worse or rejected by a therapist who brings up this topic. (F, 40)

We only deal with these problems if the patient brings them up as something important. It is a very individual and intimate topic. If the patient mentions it more or less directly, then of course I do, but as a standard question in the interview it could be too intrusive. (F, 59)

Some mental health professionals only decided to include the subject of spirituality in diagnostic or psychotherapeutic sessions if they found symptoms of psychopathology or other problems that were related to spiritual issues. In their opinion, only situations in which direct associations can be seen between spirituality and mental disorders give the mental health professional the right to work on this area of functioning:

Generally, I don’t work with spirituality because I only concentrate on clinical knowledge and experience. But there are exceptional situations, in which positive symptoms in psychotic patients may include religious content, or in other cases, excessive religiosity may seem to be a “disorder,” but it may actually be an expression of intensive mystical experiences. (M, 71)

A few mental health professionals included the issue of spirituality only in an indirect way, through conversations basically oriented at the client’s mental functioning, which would sometimes develop into conversations about his or her spirituality:

I ask patients about their values, goals and dreams, and then topics connected with spirituality may arise. (F, 39)

Some mental health professionals did not include the clients’ spirituality in the diagnosis, and a few participants did not address this topic during the therapy. According to those mental health professionals, there is no need to include clients’ spirituality into treatment since it is not relevant to the objectives of mental health care:

I don’t diagnose the patient’s relationship with God. I think this is not why they come to me. (F, 49)

Patients have mental, not spiritual problems. (M, 53)

Lack of scientific basis for the concept of spirituality, the obscurity of definitions and criteria used, and the lack of appropriate measurement instruments were the other reasons for not including spirituality in treatment. There were also some views that spirituality cannot be captured at all using psychological methods because its essence eludes the scientific cognition:

I’ve never found any convincing theories, definitions, or instruments to assess spiritual health, or any evidence that such a concept is true. (M, 45)

A few participants mentioned their limited knowledge on spirituality, their own lack of spiritual experiences, and fear as the reasons for not including clients’ spirituality in treatment. Those mental health professionals did not feel competent enough to bring up this subject during their therapeutic work, although their general opinion on applying spirituality in treatment was rather positive:

Although it is an important sphere of life, and I would like to include it more often in my therapeutic work, my little professional experience [with it] often prevents me from doing so because I don’t have enough courage. Besides, this topic was not mentioned in any of the courses I did, so I would need some guidelines how to include spirituality in psychotherapy. (F, 38)

Ethical inappropriateness was also mentioned as the reason for not including the subject of spirituality in treatment. For those mental health professionals, including spirituality in therapy would contradict the ethical principle of having a neutral attitude toward clients’ beliefs:

I don’t mention such content mostly because, in accordance with the ethical principles of psychotherapy, I cannot interfere in the person’s value system. (F, 49)

### Techniques Used to Work with Clients on Spiritual Issues (Question 5)

Two main categories of techniques used by mental health professionals when providing spiritual care to their clients were identified: (1) non-specific techniques and (2) specific techniques. Techniques that were only indirectly (implicitly) related to spirituality were termed “non-specific,” whereas those that explicitly introduced spiritual issues were categorized as “specific.”

A majority of the participants used non-specific techniques when working with the clients’ spirituality. Among the frequently mentioned techniques of this kind was the unstructured interview, which dependent on the context; issues mentioned by the client during the therapy could be utilized in treatment:

I cannot describe the techniques, but I can talk about the areas of conversation. If my patient treats her work, which is the design and manufacture of jewelry, as a specific contact with herself and her Self (the unexplored part of herself that allows her to find the meaning of life) in the therapy, I talk to her about it and identify it as her resource, and I discuss with her how she can use it to a greater extent. (F, 41)

Other non-specific techniques mentioned by the mental health professionals were usually closely related to the therapeutic approaches in which the participants had been professionally trained:

Psychodynamic interventions, life story work, dream analysis, psychodrama, visualization, psycho-drawing…a tale, a poem, a symbol. (F, 55)

The subject of spirituality quite often occurs in work on dreams, metaphors, or techniques connected with artistic expression and personal creativity (drawing, music, poetry). (M, 31)

Some mental health professionals used specific techniques when working with the clients on their spirituality. The most frequently mentioned method of this kind was using standard questions, directly asking the clients about spiritual issues. Such questions usually referred to the client’s attitude toward spirituality and religion, changes in this regard over his or her lifespan, the importance of spirituality in the client’s life, and the potential associations between spirituality and reported symptoms:

How much have spiritual or religious values shaped your life? How much did spiritual and religious values affect the problems (disorders) that have brought you to the psychotherapist? Have you been a member of a religious movement, community or group? If so, what is it and how important is it for you? (M, 66)

Among the specific techniques used were various types of meditation, which were mentioned by a few mental health professionals. Other specific techniques were work on the image of God (including symbols of God or a letter to God), contemplation, tantra, reference to religious texts, Bibliodrama, a map of forgiveness, and practicing compassion and gratitude. Only a few mental health professionals actively encouraged their clients to engage in spiritual practices (such as prayer, meditation, participation in spiritual workshops and retreats, and reading books on spirituality) or mentioned introducing prayer into the therapeutic sessions:

I inspire…and encourage them to read spiritual books, go on a spiritual retreat, participate in workshops and seminars led by spiritual and religious spiritual guides, and I teach or recommend to more interested patients specific techniques for deepening their spirituality. (F, 52)

### Sources of Knowledge and Inspiration (Question 6)

With regard to sources of knowledge and inspiration concerning the inclusion of spirituality in therapy, seven categories were extracted: (1) reading, (2) personal beliefs and practices, (3) conversation with specialists and non-specialists, (4) training sessions and courses devoted to spirituality, (5) therapeutic experiences, (6) art, and (7) theological education.

The mental health professionals acquired information on spiritual care in therapy primarily from reading books on world religions and philosophies, scientific articles, and therapeutic guidebooks. Few searched for knowledge and inspiration in scriptures and in poetry.

Another frequently mentioned source of knowledge and inspiration on including spirituality in therapy were the participants’ own personal spiritual beliefs and practices, such as meditation (especially mindfulness), contemplation, prayer, yoga, participation in formation sessions and spiritual retreats, exercises of Ignatius Loyola, tantra, or listening to Hindu satsang.

Some respondents also mentioned conversations with other specialists (e.g., as part of supervision and clinical meetings or talking to members of the clergy) and non-specialists (family and friends) as a source of knowledge and a factor motivating them to include the subject of spirituality in their therapeutic practices.

Taking part in specialist training sessions and courses (e.g., Christian integrative therapy or logotherapy) and participating in conferences on spirituality were other valuable sources of knowledge and inspiration for some participants. These mental health professionals stated that participation in such events deepened their knowledge on therapeutic techniques pertaining to spirituality and inspired them to utilize this knowledge in their therapeutic practices.

Some mental health professionals mentioned the stimulating role of their own therapeutic experiences, especially the opportunity to learn from their clients about their spiritual life. For few participants, the sources of knowledge and inspirations were art (e.g., participation in concerts and other artistic events) and theological education.

## Discussion

### Definitions of Spirituality and Its Relationship with Religion

The purpose of this study was to explore the definitions, opinions, and practices concerning spirituality among Polish mental health professionals. It can be noted that the definitions of spirituality proposed by the participants are similar to the conceptualizations presented by scholars (George et al. 2000; Hill et al. 2000). When defining spirituality, Polish mental health professionals primarily referred to the relational and transcendent characters of spirituality and perceived spirituality as an important dimension of functioning, which helps to develop a sense of meaning in life and to organize the value system. Interestingly, nearly all respondents did not equate spirituality with religion. Most mental health professionals believed spirituality to be a broader term that religion. The opinion shared by many contemporary researchers studying this issue—that spirituality and religion are considered to be distinguishable, yet overlapping constructs (Hill et al. 2000; Miller and Thoresen 2003)—was slightly less popular in this group. The polarization of spirituality and religion highlights the positive character of spirituality (as having more subjective, private, and personal characteristics) and devalues religion (seen as more formal, institutionalized, and ritual); though occasionally expressed by scholars from Western societies (Fuller 2001; Mitroff 2003), this polarization is rare in definitions proposed by Polish mental health professionals. These differences may be explained by the specifics of Polish culture, in which Catholicism has strongly influenced the pathways of spiritual development. Overall, Polish mental health professionals seem to have a quite comprehensive understanding of spirituality: more or less associated with religion, but not equated with it.

Despite these findings, it should be also noted that some participants reported difficulty in defining spirituality, pointing to its elusiveness and indefinability. The lack of definitions of spirituality or the obscurity of spiritual topics may be the important factors in the exclusion of spirituality from therapeutic practice (Souza 2002). This may be problematic, especially if the client is highly religious or spiritual, psychopathological symptoms are associated with religious or spiritual issues, or a client simply wishes to explore some spiritual or religious topics during treatment (Johnson and Hayes 2003; Weld and Eriksen 2007). The potential gap in understanding suggests that each mental health professional should take a closer look at his or her own understanding of spirituality and consider its influence on his or her attitude toward clients’ spiritual needs to prevent personal doubts or biases from harming clients (Association for Spiritual, Ethical, and Religious Values in Counseling [ASERVIC] 2009; La Torre 2002).

### Opinions and Practices Concerning Spirituality in Mental Health Care

In general, the opinions of Polish mental health professionals about including spirituality in mental health care can be described as moderately positive. Most of them stated that they included clients’ spirituality in both diagnostic and psychotherapeutic sessions. Nonetheless, we regarded the opinion of Polish mental health professionals on the inclusion of spirituality in mental health care as only moderately positive because some of them decided to work on a client’s spirituality only under certain circumstances or conditions. Most of those mental health professionals stated that they do not initiate the subject of spirituality unless the client brings it up first, or they notice that the client’s psychopathological symptoms are related to spirituality. This finding is consistent with the results of other studies, which suggest that taking a proactive approach to including clients’ spirituality in assessment, is not a standard practice among mental health professionals (Cornish et al. 2012; Coyle and Lochner 2011; Hathaway et al. 2004). For example, in a study by Crossler and Salter (2005) among clinical psychologists, some of them said that they wait for client to bring up spiritual issues on the assumption that if these were significant, the client would raise them without prompting. However, as documented by Knox et al. (2005), this assumption may be misleading. In a sample of 20 adult clients, Knox et al. (2005) noted that the participants did not raise religious or spiritual topics even though they wanted to do so out of fear of being judged or misunderstood, feelings of discomfort stemming from perceived differences with their therapists, and not knowing whether such discussions were allowed or seen as appropriate by a therapist. It should be also emphasized that not including spirituality into the initial assessment is incompatible with the ethical guidelines and code of ethics for mental health professionals (ASERVIC 2009; American Counseling Association 2014; see also Coyle and Lochner 2011; Vieten et al. 2016).

The results concerning mental health professionals’ use of techniques referring to spirituality are also worth taking a closer look. Relatively few Polish mental health professionals used specific methods directly referring to spirituality. For most participants, spiritual issues were explored solely as part of an unstructured interview, and so they depended highly on the context and the topics brought up by the client. Some mental health professionals used standard questions or other specific methods to explore spiritual topics. None of the participants mentioned using instruments to measure the spirituality of clients.

Tan (1996) distinguished two forms of integration of religion and spirituality into therapy: implicit and explicit. In the implicit form (also called “covert”)—which seems to be closer to Polish mental health professionals than the latter—therapists demonstrate respect for their clients spiritual beliefs by discussing them in an indirect way. However, they would not utilize specific spiritual techniques such as reading scriptures with a client or explicitly teaching a client the religious or spiritual exercises. According to Tan, these two approaches to the integration of religion and spirituality are not mutually exclusive but rather points along an integration continuum. For some clients, the covert approach seems to be adequate and sufficient, but for others, probably the highly religious or spiritual especially, it is likely to preclude the benefits of using more explicit techniques. This assumption is partially supported by the results of the meta-analysis of 31 spiritual therapies, which showed that explicitly teaching spiritual concepts and relating them to the clients’ situation or well-being were especially beneficial techniques for them (Smith et al. 2007). Comparison of the benefits of explicitly and implicitly integrating spirituality into mental health care needs further investigation.

Given a good understanding of the concept of spirituality and a moderately positive opinion on its inclusion in therapy among Polish mental health professionals, it seems interesting that many of them do not routinely assess clients’ spirituality, do not initiate the topic of spirituality during the diagnostic sessions, or do not apply specific techniques when working on clients’ spiritually. A number of studies have shown that the majority of mental health professionals perceived client spirituality as theoretically important, beneficial for health and relevant to treatment, yet many of them are not using spirituality in practice or only include it to a limited extent (Cornish et al. 2012; Frazier and Hansen 2009; Hathaway et al. 2004; Rosmarin et al. 2013). Several reasons have been posited to cause these discrepancies between theory and practice: mental health professionals’ own disbelief in the spiritual realm, difficulty defining and discussing spirituality, ethical concerns, lack of education on the subject, or other unknown reasons (La Torre 2002; Steen et al. 2006; Weinstein et al. 2002). For Polish mental health professionals, ethical concerns and insufficient knowledge seem to be the primary reasons. Some of our participants did not include spirituality in their own practices due to ethical issues such as lack of knowledge or experience, fear, or the risk of violating ethical principles. These concerns are not specific to Polish mental health professionals: they have been consistently reported among mental health professionals working in other cultures (Barnett and Johnson 2011; Pargament 2007; Plante 2014). Ethical concerns may be often related to insufficient knowledge on spiritual topics, which is the result of not having adequate spiritual training (Frazier and Hansen 2009; Hofmann and Walach 2011; Rosmarin et al. 2013). In our study, only a few mental health professionals declared that they participated in specialized training courses and workshops concerning spirituality. Instead, our respondents often referred to their own spiritual beliefs and practices as the sources of knowledge about and inspiration for using spirituality in therapy. Although having insight into one’s own spiritual beliefs is a very important element of preparation for therapeutic work (Daghan 2018; Vieten et al. 2016), it may fail to provide mental health professionals with the skills and competencies needed to effectively work with clients’ spirituality. In light of the lack of adequate training on spirituality, Polish mental health professionals should refer to ethical guidelines, codes of ethics and other therapeutic and scientific sources discussing the principles for including spirituality in therapy, describing basic and advanced spiritual skills and competencies of mental health professionals in this regard, and offering spiritual techniques and diagnostic measures (ASERVIC 2009; Pargament 2007; Sperry and Shafranske 2005; Vieten et al. 2016). They should also bear in mind that it is only appropriate to use spiritual interventions if they are reasonably confident that they are able to implement them in a professional way (Richards and Bergin 2005; Tan 1996). Also, they should be aware that they are obliged to obtain informed consent from a client before using any spiritual interventions in therapy (Barnett and Johnson 2011).

In addition to seeking and reading specialist literature, mental health professionals may also discuss their concerns, fears, and doubts related to raising the topic of spirituality in therapy with more trained specialists and supervisors (Richards and Bergin 2005). Moreover, considering Poles’ high level of religiosity, initiating collaboration with clergy may facilitate the acquisition of knowledge on religious and spiritual concepts, language, and scriptures that may be applied to the client’s individual situation and needs (Plante 2014; Smith et al. 2007).

### Strengths and Limitations of the Study

To our knowledge, this is one of the first studies carried out among mental health professionals from a non-Western culture concerning their definitions of spirituality and their opinions and practices around including spirituality in their own therapeutic practice. It provides deeper knowledge on the inclusion of spirituality in mental health care, with specific consideration to a culture with a high level of religiosity and religious homogeneity. Another strength of the study is the use of content analysis to explore the participants’ responses. In the case of studying spirituality, which is such an abstract phenomenon, the use of qualitative methods seems especially valuable because they allow to obtain in-depth data involving the cultural context in which the participants are immersed (Belzen and Hood 2006).

Interpreting the results, we need to remember the limitations stemming from the data collection process. First, we did not offer the respondents any definitions of spirituality. Variability in the definitions of spirituality given by our participants might have influenced to some extent their responses to questions concerning their opinions and practices around the inclusion of spirituality in therapy. However, such a solution was dictated by the purpose of the study and our willingness to investigate the respondents’ definitions, opinions, and practices together.

Second, only a quarter of the mental health professionals who received the invitation by e-mail completed the questionnaire. Presumably, the mental health professionals interested in the subject of the study were overrepresented among those who agreed to participate. Due to the relatively low response rate, we decided to not analyze the data using quantitative methods. However, it should be noted that the sample size was relatively large and that the participants were heterogeneous in terms of sociodemographic and work-related characteristics. This suggests that the collected data reflect a wide range of opinions and practices on the studied subject, making it possible to learn the arguments of both the advocates and opponents of including spirituality in mental health care. Future studies should target clients of mental health services in Poland to investigate whether mental health professionals’ practices and beliefs match clients’ spiritual needs and preferences.

## Conclusions

Our study suggests that the highly religious context of Poland has a modest impact on Polish mental health professionals’ definitions, opinions, and practices concerning spirituality. The majority of the participants in our study provided quite comprehensive definitions of spirituality, perceiving spirituality as a concept related to religion, but not identical to it. They also expressed a moderately positive opinion on including spirituality in therapy. However, some of them were afraid of violating ethical principles when working on clients’ spirituality. The majority of Polish mental health professionals limited themselves to using only non-specific methods of addressing clients’ spirituality, predominantly unstructured interviews based on context-dependent, open-ended questions. By contrast, specific spiritual techniques were used rather rarely, as were those strictly related to religion like referring to religious scriptures or praying with a client. Relatively few therapists took part in training sessions dedicated to spiritual issues.

From a practical point of view, the results of our study suggest that Polish mental health professionals need to acquire specialized training to help them provide spiritual care in a professional manner. A closer collaboration of Polish mental health professionals with other specialists (more experienced therapists, supervisors, clergy, and researchers) is also highly recommended. This cooperation may result in the development of ethical standards for including clients’ spirituality in mental health care with consideration to both the cultural specifics of Poland and the individual needs and preferences of clients (see Plante 2014).
